# Gender-specific characteristics of hypertrophic response in cardiomyocytes derived from human embryonic stem cells

**DOI:** 10.34172/jcvtr.2021.32

**Published:** 2021-05-16

**Authors:** Shiva Ahmadvand, Ali Osia, Anna Meyfour, Sara Pahlavan

**Affiliations:** ^1^Department of Stem Cells and Developmental Biology, Cell Science Research Center, Royan Institute for Stem Cell Biology and Technology, ACECR, Tehran, Iran; ^2^Cobel Darou, Tehran, Iran; ^3^Basic and Molecular Epidemiology of Gastrointestinal Disorders Research Center, Research Institute for Gastroenterology and Liver Diseases, Shahid Beheshti University of Medical Sciences, Tehran, Iran

**Keywords:** Embryonic Stem Cells, Cardiomyocyte Differentiation, Sexual Dimorphism, Hypertrophy

## Abstract

***Introduction:*** Gender-specific phenotypes of the heart were reported with respect to both physiology and pathology. While most differences were associated with the sex hormones, differential expression of genes received special attention, particularly X-Y chromosomes’ genes.

***Methods:*** Here, we compared cardiogenesis by gene expression analysis of lineage specific markers and X-Y chromosomes’ genes, during *in vitro* differentiation of XY and XX human embryonic stem cells (hESC), in a hormone-free setup.

***Results:*** Downregulation of pluripotency marker (*NANOG*) and upregulation of cardiac mesoderm and progenitor markers (*GATA4, TBX5, NKX2.5, ISL1*) was remained temporally similar in differentiating XY and XX hESCs. Isoproterenol treatment of XY and XX hESC-derived cardiomyocytes (hESCCM) induced hypertrophy in a sex-specific manner, with female cardiomyocytes showing response at higher isoproterenol concentration and a later time point of differentiation. Interestingly, *KDM5C* as an X-linked gene, was markedly upregulated in both hypertrophied male and female cardiomyocytes.

***Conclusion:*** Collectively, our results indicated a temporally identical cardiogenesis, but more susceptibility of XY hESC-CM to hypertrophic stimulus in a hormone-free condition.

## Introduction

Gender-specific phenotypes of cardiovascular system have been reported with respect to both physiology and pathology.^1,2^ Baseline cardiac physiology differs in healthy men and women, with latter showing better diastolic function and preserved systolic function by aging. Cardiac electrophysiology displays sexual dimorphism as well, where the repolarization phase is longer in females making them more prone to arrhythmias.^3^ Interestingly, these gender-specific characteristics exist at cellular level, too. The Ca^2+^ current (I_Ca,L_ density) was higher in the canine female cardiomyocytes independent of estrogen.^4^ Female rat cardiomyocytes showed weaker and slower contractions compared to male due to different Ca^2+^ stores, while other components of excitation-contraction coupling machinery revealed sex bias as well.^5^ *KCNH2*, *KCNE1*, *KCNJ4*, *KCNA4*, *KCNIP2*, *KCNA5*, *KCNJ11*, *GJA1* and *PLN* showed lower expression in female compared to male human cardiomyocytes.^6^ While most of these sex-related differences have been associated to the function of sex hormones,^7^ molecular biology studies have assigned hormone-independent role to the differential expression of genes from which many are located on X-Y chromosomes. ^8^ Some of these genes were identified such as *USP9Y*, *RPS4Y1*, *DDX3Y*, *XIST*, *TIMP1*, *UTY*, *ZFY*, *PRKY*, and *JARID1D*,^8,9^ however, their function in the sexual dimorphism has not been fully elucidated. Particularly, very few information exists on the sex-related differences in cardiac development.^10^ Llamas and colleagues could identify a contributory role for Y chromosome in the loci governing cardiomyocyte size by crossing Y chromosomes from two different mouse strains onto a single background strain.^11-13^ The observed effect on cardiac cell size was independent of testosterone.^14^ Furthermore, they profiled expression of Y chromosome genes in the heart of two mouse strains with various cardiomyocytes size and observed different expression of *Jarid1d*, *Uty*, *Ddx3y*, and *Eif2s3y*.^15^ The function of UTY was further studied by assessing its expression profile during cardiogenic differentiation of embryonic stem cells as well as in UTY/UTX knockout mice.^16-18^ Our group could also identify differential expression of some MSY genes compared to their X counterparts, particularly at cardiac mesoderm stage, during *in vitro* differentiation of human embryonic stem cells (hESC) into cardiomyocytes.^19^

Post-natal cardiac development mostly involves the cardiomyocyte growth rather than increase in the cell number. The cardiomyocyte hypertrophy is primarily a dynamic and adaptive response in the normal state (physiologic). But it might turn to adverse remodeling (pathologic) in response to biomechanical stress and/or neurohormonal stimuli.^20^ Although it is considered a beneficial response, prolonged hypertrophy may cause heart failure and sudden cardiac death. Similar to many other cardiovascular phenotypes, pathological hypertrophy shows gender-related characteristics^21^ Interestingly, all various types of hypertrophic stimuli including pressure overload, volume overload, and hormones resulted in gender-specific phenotypes with male developing more severe disease symptoms. Male hypertrophic hearts showed lower contractility, more fibrosis, higher mortality rate and more prone to heart failure.^21^ Proteomic analysis of hypertrophic hearts identified the higher expression of cytoskeletal proteins in females but mitochondrial proteins in males, suggesting the activation of different signaling pathways in response to same pathological stimulus.^22^ Although these phenotypic differences have been greatly attributed to estrogen, supplementation of ovariectomized female rats with 17β estradiol did not fully rescue the hypertrophic remodeling.^23^

Most of the present knowledge on sex-related differences of cardiac physiology and pathology has been obtained using animal models. However, the translation of these findings into human would be challenging due to the inter-species differences. In order to study molecular regulator(s) of sex-specific cardiac phenotypes, especially during cardiac development, a hormone-free *in vitro* model of human cardiomyocytes would be of utmost importance. Cardiogenic differentiation of hESC provides a versatile tool for such studies due to the potential for simulating cardiac development and sample collection at different developmental stages *in vitro* in a hormone-free setup.^24,25^ In the current study, we evaluated the expression profile of some X/Y chromosome genes as well as cardiac specific genes during cardiogenic differentiation of XX and XY hESCs, in order to identify developmental differences and their relation to differential expression of sex-specific genes. Furthermore, we used an *in vitro* isoproterenol-induced hypertrophy model in XX and XY hESC-derived cardiomyocytes, as a platform to study the role of sex chromosome genes in sex-specific hypertrophic characteristics.

## Materials and Methods

### Expansion and cardiogenic differentiation of hESCs

Two hESC lines (female Royan H17 [RH17] and male Royan H6 [RH6] ^26^) were expanded in static suspension culture. Induction of cardiomyocyte differentiation was performed by incubating 5-day-old hESC size-controlled aggregates (average size: 175 ± 25 µm) for 24 h in differentiation medium composed of RPMI1640 (31870-022, Gibco) supplemented with 2% B27 minus vitamin A (12587-010, Gibco), 2 mM L-glutamine (Gibco), 0.1 mM β-mercaptoethanol (Sigma-Aldrich), 1% non-essential amino acids (Gibco), and 12 mM of the small molecule (SM) CHIR99021 (CHIR; 041-0004, Stemgent). After 24 h, the aggregates were washed with Dulbecco’s phosphate buffered saline (DPBS) and were maintained in fresh differentiation medium without SM for 1 day. At day 2, the medium was exchanged for new differentiation medium that contained 5 mM IWP2 (3533, Tocris Bioscience), 5 mM SB431542 (S4317, Sigma-Aldrich), and 5 mM purmorphamine (Pur; 04-0009, Stemgent) supplemented with B27 minus insulin. The aggregates were cultured for 2 days in this medium. After washing with DPBS, fresh differentiation medium was added to the culture and refreshed every 2–3 days. Samples were collected at different time points (days 0, 1, 3, 6, and 12) of differentiation from three biological replicates for further analysis.

### Immunofluorescence staining

The pluripotency of RH17/RH6 hESCs and cardiogenic differentiation of RH17/RH6-derived cells were evaluated using immunofluorescence staining against OCT4, NANOG and cardiac troponin T (cTNT), respectively. hESC aggregates and beating spheroids at day 12 of differentiation were collected, washed twice with PBS and dissociated into single cells with 0.05% trypsin-EDTA (25300-054; Gibco) at 37°C for 4–5 minutes. Individualized cells were cultured on Matrigel-coated 4-well plates (354248, Corning). After 2 days, the attached cells were washed once with PBS, fixed with 4% (w/v) paraformaldehyde at room temperature for 15 minutes, washed once with washing buffer (PBS/0.1% Tween 20), permeabilized with 0.2% Triton X-100 in PBS for 15 minutes, and blocked with 5% (v/v) bovine serum for 1 h. Primary antibodies diluted in blocking buffer (PBS/0.2% Triton X-100/5% bovine serum) (1:100) were added to the cells and incubated overnight at 4°C. After incubation, the cells were washed three times (5 minutes each) with washing buffer. Secondary antibodies diluted in blocking buffer (1:500) were added to the cells and incubated for 1 h at room temperature. The cells were subsequently washed three times with washing buffer. The nuclei were counterstained with 4′,6-diamidino-2-phenylindole (DAPI; D-8417, Sigma-Aldrich) and photographed using an Olympus IX71 microscope equipped with a DP72 digital camera. The primary and secondary antibodies are listed in Supplementary Table 1.

### Quantitative real time PCR

RNA isolation was carried out using TRIzol reagent (Invitrogen,15596018, USA) and RNeasy micro kit (QIAGEN, 74004, USA) according to themanufacturer’s protocol. Any potential DNA contamination was removed by treating theextracted RNA with RNase-free DNase (Takara, Japan). The resultant RNA wasreverse-transcribed into cDNA using Easy cDNA Synthesis Kit (A101161, Pars Tous Biotechnology) and then diluted to 25 ng/μL for quantitative real-timePCR (qRT-PCR) using *Applied Biosystems*^TM^ StepOne^TM^ *Real*-*Time PCR*System (Fischer scientific, Canada). *GAPDH*was used as the housekeeping gene. All experiments were performed from three biological replicates. All primers were designed using PerlPrimer (v1.1.21., SOURCEFORGE) and are listed in Supplementary Table 2.

### In vitro hypertrophy induction

To induce hypertrophy, RH17/RH6-derived cardiomyocytes were treated with 10 or 30 μM isoproterenol (ISO, Sigma-Aldrich) for 48 hours ^27,28^ at day 13 or 21 of differentiation. The hypertrophic response was evaluated by analysis of cell area and expression profile of fetal genes including *MYH6*, *MYH7*, *ATP2A2*, *NPPA*, *NPPB* in RH17/RH6-derived cardiomyocytes. To evaluate cell area, cardiomyocytes were fixed in 4% paraformaldehyde, permeabilized, blocked and stained with anti-cTNT antibody. Images were acquired using an Olympus IX71 microscope equipped with a DP72 digital camera. Cell areas were measured using image J software (1.46r Wayne Rasband, USA). For gene expression analysis, samples were collected from three biological replicates of untreated and ISO-treated cardiomyocytes 72 h after hypertrophy induction and subjected to quantitative real time PCR.

### Statistical analysis

Data are presented as mean ± SEM from three biological replicates, otherwise stated. Statistical significance was tested by using two-tailed unpaired student’s t-test and one-way and two-way ANOVA in Graphpad Prism8 software (Graphpad Software, USA). ^∗^*P* < 0.05, ^∗∗^*P* < 0.01, and ^∗∗∗^*P* < 0.001 indicated as statistically significant.

## Results

### Similar temporal pattern of in vitro cardiogenesis in male and female cells

**Figure 1:**
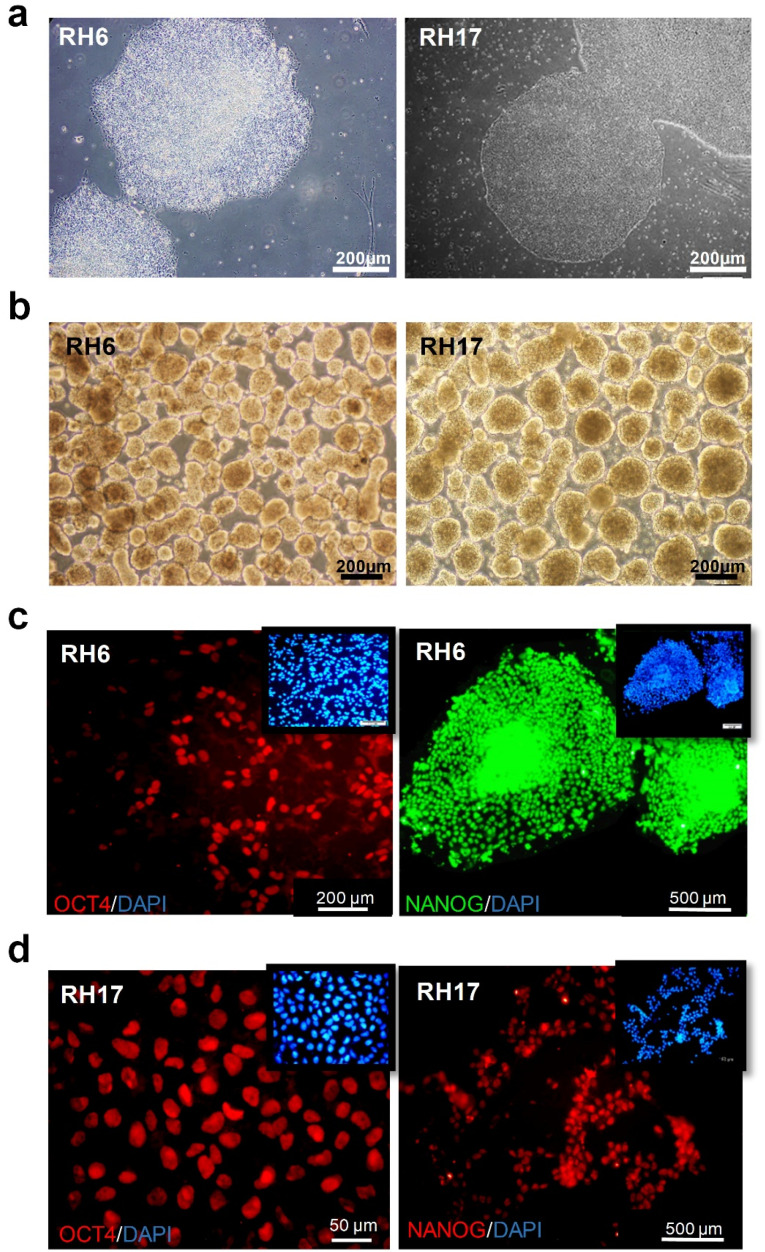
Characterization of RH6 and RH17 human embryonic stem cells (hESCs). (a) Microscopic image of adherent hESCs RH6 (left) and RH17 (right) on matrigel. (b) Microscopic image of spheres when the average size was 175 ± 25 µm. (c, d) Immunostaining analysis of RH6 and RH17 hESCs for (NANOG and OCT4). Nuclear staining was performed using DAPI

**Figure 2:**
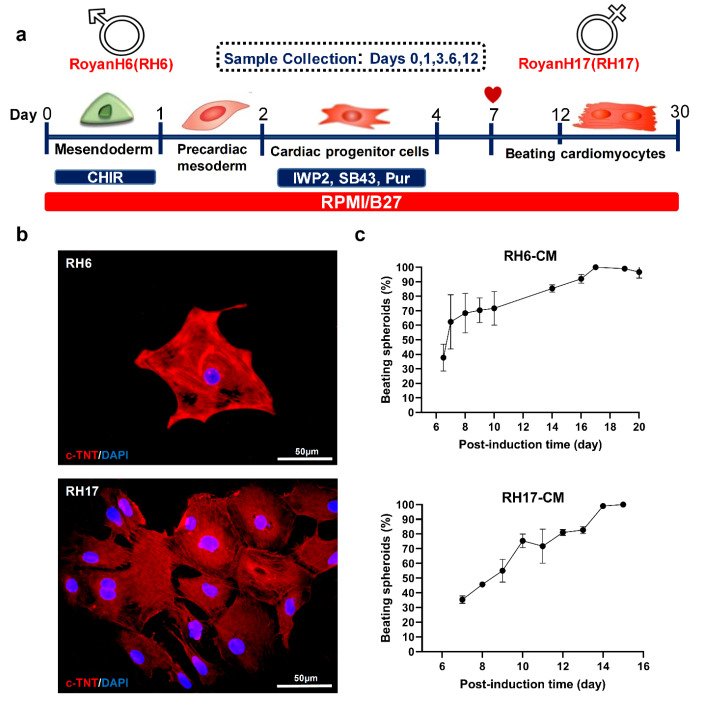
Schematic representation of the cardiac differentiation protocol in a static suspension culture. (a) hESCs were induced to cardiac mesoderm using a cocktail of the small molecules (SMs) including CHIR (CHIR99021): Wnt inducer and to cardiac progenitor cells using SB431542: Inhibitor of transforming growth factor-β, Pur (Purmorphamine): a sonic hedgehog (SHH) agonist and IWP2: Wnt antagonist. Samples were collected at pluripotency (D0), mesendoderm (D1), cardiac mesoderm (D3), cardiac progenitor cell (D6) and cardiomyocyte (D12) stages in both RH6 and RH17 differentiating hESCs for further analysis. (b) Immunostaining of cardiomyocyte-specific cytoskeletal protein cTNT; scale bar, 50 μm. Nuclear staining was performed using DAPI. (c) Beating rate of RH6 and RH17 hESC-CM up to day 20 of cardiac differentiation

Human pluripotent stem cells provided a versatile source for generation of most cell types of human body *in vitro*. Furthermore, studies of biological processes, physiology and pathology were facilitated in both male and female using *in vitro* differentiation of these pluripotent stem cells. Taking advantage of this emerging technology, cardiac development was compared in male and female by investigating various developmental stages during cardiogenic differentiation of male RH6 and female RH17 hESCs. Both embryonic stem cell lines were expanded and formed colonies of stem cells (Figure 1a). Before directed differentiation, hESCs were further expanded in static suspension culture, thus generating cell aggregates with a size range of 175 ± 25 µm diameter (Figure 1b). RH6 and RH17 hESCs were all stained positive for two pluripotency markers, OCT4 and NANOG (Figure 1c and d). These cells were subjected to cardiogenic differentiation and the resulting cells were collected for gene expression analysis at day 0 corresponding to pluripotency stage, day 1 to mesendoderm, day 3 to cardiac mesoderm, day 6 to cardiac progenitor cells and day 12 to cardiomyocytes, as were previously defined on the established protocol of our group (Figure 2a). ^24^ Both RH6- and RH17-derived cardiac cells (RH6-CM and RH17-CM) were stained positive for cardiomyocyte-specific cytoskeletal protein, cTNT (Figure 2b), and all differentiated cells showed spontaneous beating at day 17 and 14 post-differentiation, respectively (Figure 2c and Supplementary Video S1). It is worth noting that beating started at the same day (day 7 post-differentiation) in both XY and XX cardiomyocytes comprising >30% of differentiated cells. The beating population was increased in both male and female cells until reaching 100%, with RH6-CM showing a 72 hours delay (Figure 2c).

**Figure 3:**
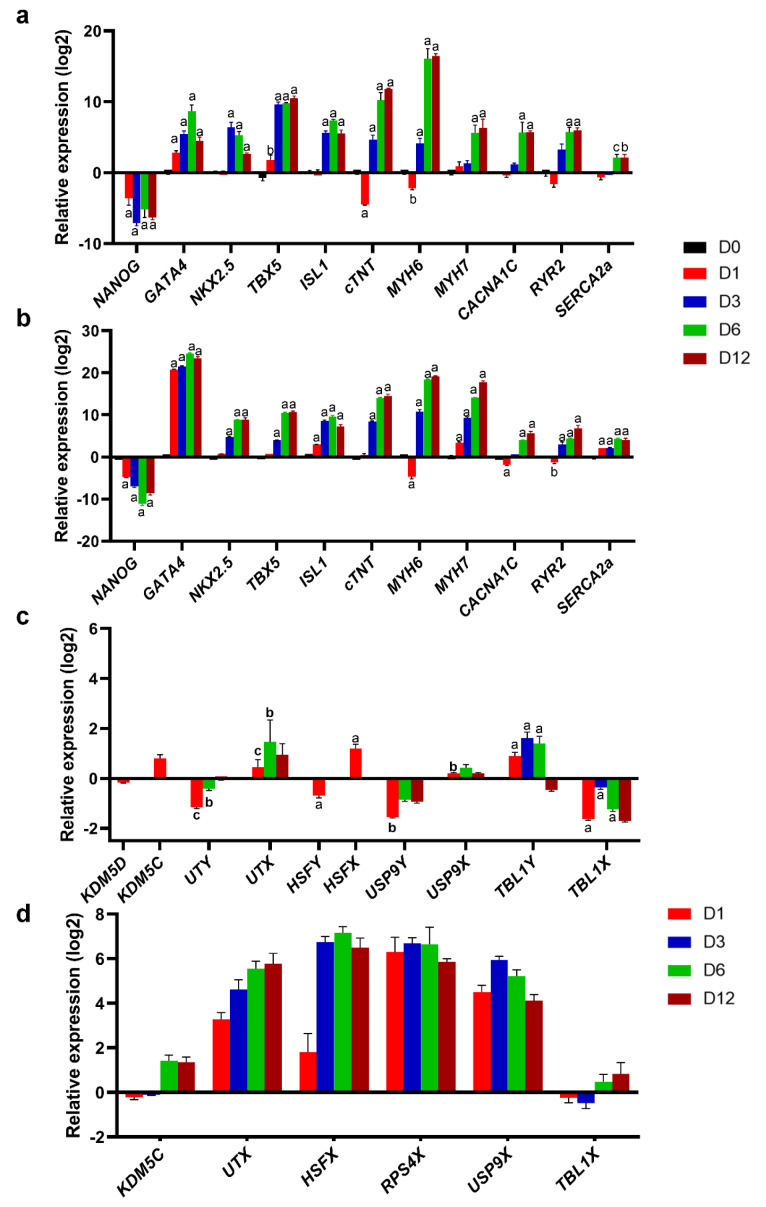
Transcriptional expressions of cardiac specific and sex-specific chromosomes genes at different stages of cardiac differentiation. D0, D1, D3, D6, and D12 which represent pluripotency, mesendoderm, cardiac mesoderm, cardiac progenitor cells, and cardiomyocyte, respectively. (a, b) The expression patterns showed downregulation of pluripotency marker (*NANOG*) in differentiating cells, increased expression of progenitor markers (*GATA4, TBX5, NKX2.5, ISL1*) at day 3 until day 12, upregulation of contractile apparatus genes (*cTNT, β-MHC, α-MHC*) at day 6 and 12 and upregulation of Ca^2+^ handling genes (*RYR2, SERCA2a, CACNA1C*) at day 6 and 12. Expression of X chromosome genes (*KDM5C, UTX, HSFX, USP9X, TBL1X*) and Y chromosome genes (*KDM5D, UTY, HSFY, USP9Y, TBL1Y*) of differentiating XY and XX hESCs (c, d). The expression pattern in some Y chromosome genes showed an opposite pattern compared to their X counterparts in differentiating RH6 hESC. The relative expressions of all stages were compared to D0. All experiments were performed in two (3b and 3d) or three biological replicates (3a and 3c), and data were presented as mean ± SEM, *P < 0.05, **P < 0.01, ***P < 0.001

The progression of cardiogenic differentiation was compared in male and female cells by evaluating the important marker(s) of each stage. As shown in Figure 3a and b, *NANOG* was downregulated in differentiating cells of both lines as early as day 1 and remained downregulated over all other stages. On the other hand, *GATA4* was upregulated starting at day 1 and persisted until day 12, suggesting the same efficiency of progression into cardiac mesoderm in both male and female cells. Furthermore, upregulation of *NKX2.5*, *TBX5* and *ISL1* similarly happened at day 3 and persisted until day 12, showing a similar efficiency of cardiac progenitor cells generation with respect to gene expression level in both male and female differentiating cells (Figure 3a and b). The highest expression of *cTNT*, *MYH6* and *MYH7* was observed at day 6 corresponding to cardiac progenitor cell and day 12 for the cardiomyocyte stage. Interestingly, the gene expression of these cardiomyocyte-specific markers i.e. *cTNT*, *MYH6* and *MYH7*was increased during cardiac mesoderm stage on day 3. However, this was not the case for upregulation of L-type Ca^2+^ channel gene, *CACNA1C*, which started at day 6 corresponding to cardiac progenitor stage. Similar pattern was observed in both XY and XX hESC-CMs, indicating later expression onset of this ion channel in more cardiac committed cells (Figure 3a and b). This result further confirmed similar efficiency of differentiation progression in both male and female cells. Maximal expression of *RYR2* and *SERCA2a* was observed at days 6 and 12, indicating progression into a more organized intracellular Ca^2+^ stores of cardiomyocytes. Altogether, the gene expression analysis of cardiogenic differentiation in both XY and XX cells showed a similar temporal characteristics of cardiac cells development.

### Alterations of X-Y chromosome genes’ expression during in vitro cardiogenesis

Sex chromosome genes were always considered to play role in sex determination. However, they might also contribute in other organs development due to the presence of sexual dimorphism in multiple tissues of human body. RH6 and RH17 hESCs were interrogated with respect to the expression of X-Y chromosome genes during cardiomyocyte induction. As shown in Figure 3c, some Y chromosome genes showed a totally opposite expression pattern compared to their X counterparts in differentiating RH6 hESC. While *KDM5D*, *UTY*, *HSFY* and *USP9Y* were downregulated by promoting to mesendoderm stage, their X homologues were upregulated at the same time during cardiomyocyte differentiation. On the other hand, *TBL1Y* was upregulated at days 1, 3 and 6. While TBL1X was downregulated. These findings suggest that there might be a role for X-Y chromosome genes in cardiomyocyte development. We previously showed that TBL1Y knockdown in differentiating RH6-hESCs prior to cardiac mesoderm stage, delayed their cardiomyocyte generation and decreased the frequency of contractions in resulting cardiac cells. ^19^ As shown in Figure 3d, except *KDM5C* and *TBL1X*, other X chromosome genes including *UTX*, *HSFX*, *RPS4X* and *USP9X* were upregulated by initiation of cardiogenic differentiation in RH17 hESC and persisted all over this process. However, *KDM5C* and *TBL1X* were initially downregulated during mesoderm differentiation and then upregulated by further commitment toward cardiomyocyte (Figure 3d).

### Various sensitivities of male and female cardiomyocytes to hypertrophic stimulus

**Figure 4:**
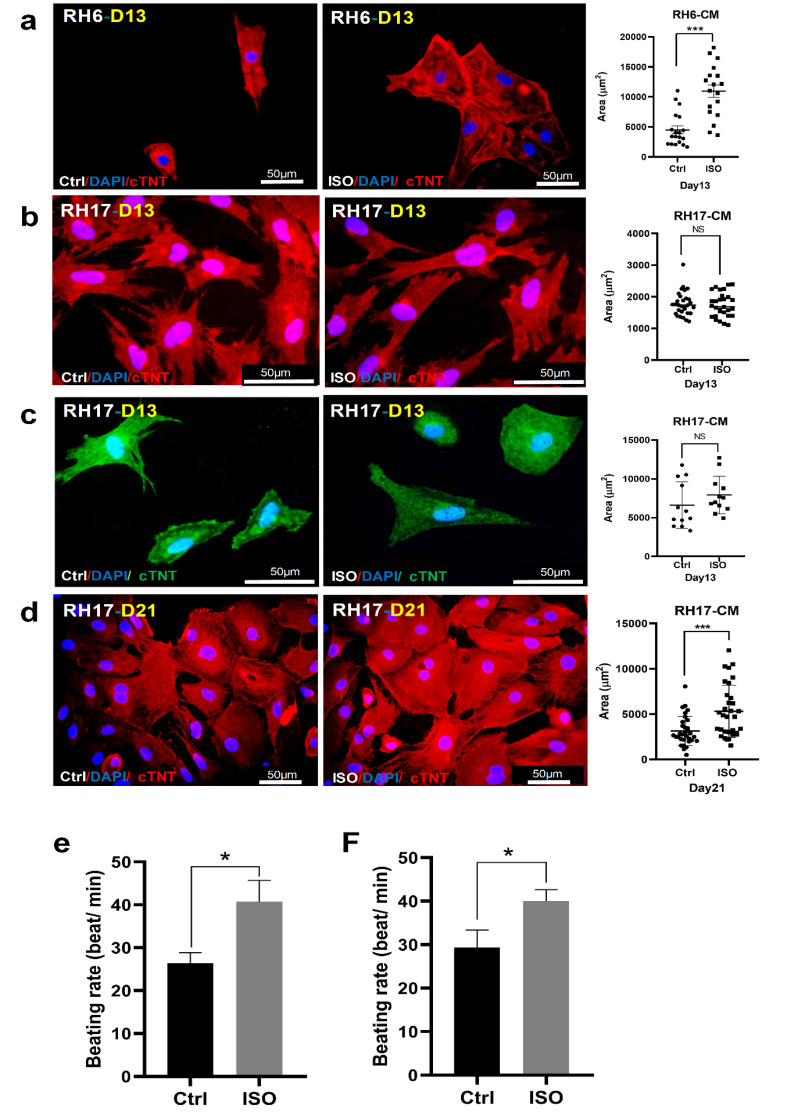
Hypertrophy induction and evaluation of area size in RH6 and RH17 cardiomyocytes. Immunostaining was performed for three different treated groups; (a) The RH6 cardiomyocytes treated at Day13 of differentiation-48h-10µM ISO showed a significant increase in area size compared to control cells (untreated). (b, c) The RH17 cardiomyocytes treated at Day13 of differentiation-48h-10µM ISO and Day13 of differentiation-48h-30µM ISO showed no significant difference in area size compared to control cells (untreated). (d) The RH17 cardiomyocytes treated at Day21 of differentaition-48h-30µM ISO showed a significant increase in area size compared to untreated cells. All data were presented as Mean ± SEM, ***P < 0.001, *NS*: *not significant*

As pathological hypertrophy reveals sex-related characteristics, hypertrophy induction and corresponding phenotypes were compared in RH6- and RH17-CM. Initially, both RH6- and RH17-CM were subjected to 10 µM isoproterenol (ISO) at day 13 of differentiation for 48 h. While cell area was substantially increased in RH6-CM after ISO treatment, RH17-CM did not show any difference in cardiomyocytes area post-treatment (Figure 4a and B). Although RH17-CM were subjected to higher concentration of ISO (30 µM) at day 13 for 48 h, hypertrophy response was not observed with respect to cardiomyocyte size (Figure 4c). Female cardiomyocytes were kept longer in culture and treated with 30 µM of ISO at day 21 of differentiation for 48 hours. As shown in Figure 4d, cardiomyocytes area was markedly increased in ISO-treated cells. These results suggest that sex-related differences in hypertrophy response might be due to various sensitivities of β1-adrenergic receptors or downstream signaling pathway in male and female cardiomyocytes. Both hypertrophied RH6- and RH17-CM showed substantially higher beating frequencies (Figure 4e and f).

**Figure 5:**
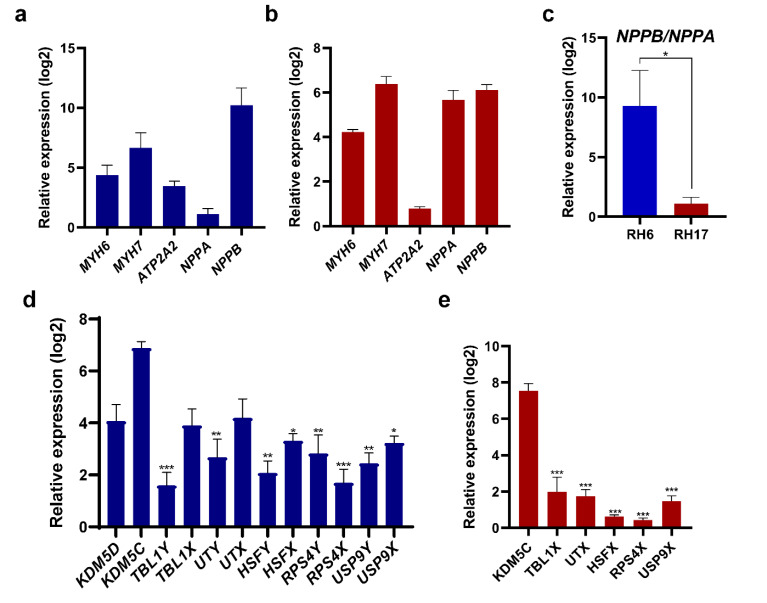
Upregulation of X-Y chromosome genes in RH6 and RH17 ISO-treated cardiomyocytes. (a, b) Upregulation of fetal genes that include *MYH6, MYH7, ATP2A2, NPPA, NPPB* in ISO-treated cells compared to control cells (untreated). (c) The *NPPB/NPPA* ratio showed a significant increase in hypertrophied RH6-CM compared to RH17-CM. (d, e) Upregulation of X-Y chromosome genes in hypertrophied RH6- and RH-17-CM. The relative expression of all genes were compared to KDM5C in both hypertrophied male and female cardiomyocytes. All experiments were performed in two biological replicates, and data are presented as mean ± SEM, *P < 0.05, **P < 0.01, ***P < 0.001

In addition to the cell size, the expression of some members of fetal genes program was evaluated in ISO-treated RH6- and RH17-CM. While upregulation of *MYH6*, *MYH7*, *ATP2A2*, *NPPA* and *NPPB* was evident in both hypertrophied male and female cardiomyoctes (Figure 5a and b), the *NPPB*/*NPPA* ratio was 6-fold higher in hypertrophied RH6-CM, suggesting a more significant hypertrophy response (Figure 5c). Furthermore, the expression of X chromosome genes (*KDM5C*, *TBL1X*, *UTX*, *HSFX*, *USP9X*) was higher compared to their Y homologues in hypertrophied RH6-CM (Figure 5d). Interestingly, *KDM5C* showed a marked upregulation in both hypertrophied RH6- and RH17-CM compared to other X chromosome genes (Figure 5e). This finding suggests an important role for KDM5C, as an X chromosome associated gene, in hypertrophy response of both male and female cardiomyocytes. However, this result should be further evaluated by *KDM5C* knockdown or knockout studies.

## Discussion

The cardiovascular system shows sex-related physiological and pathological phenotypes. ^1,2^ Not only the susceptibility differs, but also the severity of disease displays male/female specificities.^29-31^ While sex hormones have shown to underlie majority of these phenotypic differences, ^2^ but they were still present in gonadectomized animals.^4^ Thus, there have been always questions on the possibility of subcellular disparities, independent of hormone effects on intracellular processes. Particularly, differential gene expression gained high attention, considering the fact that male/female have different sets of sex chromosome associated genes. By emergence of advanced technologies, there have been some attempts to identify potential differences at gene expression level. RNA sequencing of adult human heart samples obtained from 46 post-mortem healthy individuals, showed sexual dimorphism in the expression of genes mostly related to sex chromosomes as well as autosomal genes associated to inflammatory response. ^32^

By now, there were very few studies on healthy human heart due to difficult access to this tissue. And the majority of reports are on samples from various cardiovascular diseases obtained from patients during vascular or surgical interventions. Particularly, comparative developmental studies of cardiovascular system between male and female, is challenging due to sampling issues from developing heart or vasculature at several developmental stages. And most importantly, subcellular studies from human samples are impossible in hormone-free context. Thus, the development of a system that provides access to human cardiovascular tissue, at various developmental stages, in a hormone-free setup, is of utmost importance. *In vitro* differentiation of human pluripotent stem cells could supply all the aforementioned requirements. In this study, male and female hESCs were differentiated into cardiomyocytes and the expression of some stage-specific genes of cardiomyocyte development as well as sex chromosome genes, were evaluated. Gene expression analysis is used as cell identity and stage identifier in developmental studies. Leaving the pluripotency and obtaining mesoderm and cardiac mesoderm identity, were temporally identical in both XY and XX hESCs, as reflected in the expression of their specific markers genes. Furthermore, cardiomyocyte generation from the early cytoskeletal protein genes’ expression to the later Ca^2+^ channels upregulation (*CACNA1C*,* RYR2*,* SERCA2a*), showed the same temporal pattern. Thus, cardiomyocyte differentiation did not differ between XY and XX hESCs, based on the expression of selected stage-specific markers. However, this might not reflect in the similar temporal characteristics of whole organ development or generation of a functional organ. Furthermore, this result has to be verified in more XY and XX hESC lines as well as evaluating expression analysis of more stage-specific genes. However, to the best of our knowledge, this is the first evidence on the temporal characteristics of development of human male and female cardiomyocytes, which became possible by *in vitro* differentiation of hESCs. This methodology and finding open a whole new path toward developmental studies of male and female hearts. A recent study on sex-specific transcriptome and proteome of embryonic and adult heart showed that these sex-related differences are predominately governed by post-transcriptional processes, resulted from both hormonal and sex chromosomes’ gene products, and occurred prior to gonad formation.^33^ Thus, a comprehensive study at all levels of transcriptional and post-transcriptional mechanisms would be of utmost important to elucidate all aspects of sex-related cardiovascular developmental phenotypes. The observed changes in the expression level of X-Y chromosome genes over the period of cardiogenic differentiation, suggested their contribution in this developmental process. *TBL1Y* knockdown in differentiating XY hESCs at mesoderm stage, altered differentiation process temporally and resulted in generation of functionally impaired cardiomyocytes.^19^ Furthermore, the altered expression of a Y chromosome gene*,KDM5D*, during *in vitro* cardiomocyte differentiation resulted in impaired generation of cardiac cells.^34^ However, further functional analysis of every single X-Y chromosome gene with differential expression over cardiogenic differentiation, as well as double X-Y homologous genes knockout, are required to draw a clearer image of their role in heart development. Gaining more knowledge on the contribution of X-Y chromosome genes in the development of cardiovascular system, would help in better management of cardiovascular disorders.

The sex-associated phenotypes of cardiovascular diseases have been thoroughly discovered in clinical studies.^30^ Our observation of sex-specific response to hypertrophy stimulus provided a novel insight on the mechanism of its incidence at cellular level. Female hESC-CM responded to higher concentration of ISO stimulation at a later time point of cardiomyocyte differentiation. This might suggest lower sensitivity of β1 adrenergic receptors in *in vitro* differentiated female cardiomyoctes. Moreover, there is also possibility for lower number of β1 adrenergic receptors in earlier female cardiomyocytes which forced us to induce hypertrophy at later stages. However, both hypothesis needs further experimental evidences. Cheng et al used 30 µM ISO for hypertrophy induction in both XY and XX hESC-CM and did not compare their responsiveness to hypertrophy stimulus.^28^ Moreover, a marked difference was observed in the *NPPB*/*NPPA* ratio in hypertrophied male compared to female hESC-CM in the current study, further suggesting a more significant subcellular responsiveness in male cardiomyocytes. This finding might reflect as sexual dimorphism of hypertrophy development in human heart.^21^ Sex chromosome genes can be considered as one of the sources of subcellular differences in male and female. Thus, the expression of some of X-Y chromosome genes were evaluated in hESC-CM. While both Y- and X-related genes were upregulated in hypertrophied male hESC-CM, *KDM5C* expression was markedly higher compared to its Y homologue, *KDM5D*, as well as other evaluated genes. This *KDM5C* significant upregulation occurred in hypertrophied female hESC-CM, too. *KDM5C* encodes lysine-specific demethylase 5C in humans which contributes in transcriptional regulation and chromatin remodeling. Alterations of lysine methylation throughout histones impair normal gene expression and underlie several pathological conditions such as hypertrophy.^35^ The hypertrophic cardiomyocytes showed an altered pattern of methylated histone lysine in favor of disease phenotype when evaluated by genome-wide ChIP.^36^ Furthermore, *KDM4A* upregulation has been reported in patients with hypertrophic cardiomyopathy.^35^ Mice harboring heart-specific knockout of Lysine-specific demethylase 4A did not develop cardiac hypertrophy when subjected to transverse aortic constriction.^35^ Another lysine demethylase, KDM3C, has been also associated to cardiomyocyte hypertrophy response, where its knockdown inversely impacted angiotensin II-induced fetal gene program and cell growth.^37^ KDM5C might also impacted hypertrophy-related gene program, however it should be further investigated by loss of function experiments.

## Conclusion

Sex chromosomes-related genes, such as Y chromosome-linked *TBL1Y*, might underlie male/female-specific characteristics of heart development. Furthermore, it may provide a better insight into treatment strategies applied for male or female. In addition, *KDM5C* might be introduced as a novel target in clinical management of hypertrophy response.

## Acknowledgement

We would like to thank Hassan Ansari for his assistance in cardiomyocyte differentiation.

## Competing interest

Shiva Ahmadvand declares that she has no conflict of interest. Ali Osia declares that he has no conflict of interest. Anna Meyfour declares that she has no conflict of interest. Sara Pahlavan declares that she has no conflict of interest.

## Ethical approval

This article does not contain any studies with human participants or animals performed by any of the authors.

## Funding

This work was supported by a grant from National Institute for Medical Research Development (NIMAD, #964040).

## 


Supplementary file 1 contains Table S1 and Table S2.

Supplementary file 2 contains Video S1.
