# Supplementary material for: Gender-specific characteristics of hypertrophic response in cardiomyocytes derived from human embryonic stem cells
Source: J Cardiovasc Thorac Res. 2021 May 16;13(2):146–55. doi: 10.34172/jcvtr.2021.32 (PMC8302890; doi:10.34172/jcvtr.2021.32)
Supplement: Supplementary file 1 — contains Table S1 and Table S2. [file jcvtr-13-146-s001.pdf]

Gender-specific characteristics of hypertrophic response in cardiomyocytes derived from human embryonic stem cells

Shiva Ahmadvand[1], Ali Osia[2], Anna Meyfour[3], Sara Pahlavan[1*]

[1]Department of Stem Cells and Developmental Biology, Cell Science Research Center, Royan Institute for Stem Cell Biology and Technology, ACECR, Tehran, Iran

[2]Cobel Darou, Tehran, Iran

[3]Basic and Molecular Epidemiology of Gastrointestinal Disorders Research Center, Research Institute for Gastroenterology and Liver Diseases, Shahid Beheshti University of Medical Sciences, Tehran, Iran

Supplementary file 1

Table S1. List of primary and secondary antibodies used in the study

Antibody	Cat.number	Company
NANOG antibody	AF2729	R&D systems
Anti-goat antibody	A-11055	Invitrogen
OCT4 antibody	sc-5279	Santa Cruz biotechnology
Anti-mouse antibody	A-11020	Invitrogen
Anti-Cardiac Troponin T antibody	ab64623	Abcam
Anti-goat antibody	A21223	Invitrogen

Table S2. List of primers used in the study

Gene	Forward sequence(F)<br>Reverse sequence(R)
GAPDH	F: CTCATTTCCTGGTATGACAACGA
	R: CTTCCT CTT GTG CTC TTG CT
NANOG	F: AAAGAATCTTCACCTATGCC
	R: GAAGGAAGAGGAGAGACAGT
NKX2.5	F: CCCACGCCCTTCTCAGTCAA
	R: GTAGGCCTCTGGCTTGAAGG
GATA4	F: CCT GTC ATC TCA CTA CGG
	R: GCT GTT CCA AGA GTC CTG
TBX5	F: CGATCACAGATACAAATTCGC
	R: CAGGTGGTTGTTGGTGAGC
ISL1	F: TACAAAGTTACCAGCCACC
	R: GGAAGTTGAGAGGACATTGA

SERCA2a	F: CATCAAGCACACTGATCCCGT
	R: CCACTCCCATAGCTTTCCCAG
CACNA1C	F: AATCGCCTATGGACTCCTCTT
	R: GCGCCTTCACATCAAATCCG
RYR2	F: GGCAGCCCAAGGGTATCTC
	R: ACACAGCGCCACCTTCATAAT
MYH6	F: ATTGCTGAAACCGAGAATGG
	R: CGCTCCTTGAGGTTGAAAAG
MYH7	F: GGCAAGACAGTGACCGTGAAG
	R: CGTAGCGATCCTTGAGGTTGTA
TNNT2	F: ATGATGCATTTTGGGGGTTA
	R: CAGCACCTT CCTCCTCTCAG
ATP2A2	F: TCACCTGTGAGAATTGACTGG
	R: AGAAAGAGTGTGCAGCGGAT
NPPA	F: CGAGGAAGTCACCATCAAACCAC
	R: TAGGGACAGACTGCAAGAGG
NPPB	F: TCCTGCTCTTCTTGCATCTGGCTT
	R: AAATGGTTGCGCTGCTCCTGTAAC
KDM5D	F: TGCATAAGAAAGTCACATGC
	R: TGGTTGTCTTAGTGCAGG
KDM5C	F: AGGAAGAGCTGGAGGAGGAGACTGG
	R: CGGTTCCAAGCCATTCTGGTTCT
TBL1Y	F: CTGATGGCTCTGTGTGTGTTC
	R: TATTTGGAGCTGATAGAATTGTGTTT
TBL1X	F: GCCGCCATTCGCCACCGA
	R: CTCCGAACCTCCCTCCAGCCG
UTY	F: AGTAGACCAAACCAAGGCCA
	R: GGAGTCTGACTTCTTTTGCCA
UTX	F: GAGGTTGAAGTGACCTAATTGACA
	R: CCTCTTTGGGTTCGTGAGAT
HSFY	F: TTCATGGGATGAGAATGGAAC
	R: GAAAGGTGGCTAGAAAGGCAG
HSFX	F: AACGGCGTCAATGCACTTTCA
	R: TGTCCTCCTCCTGATGTCTTGGTGC
RPS4Y	F: AGGAAGATTACTGTGGGAGTG
	R: GTATCGTTCACCTTGATGAC
RPS4X	F: GGTCCTCTTTCCTTGCCTAACGCA
	R: CACTCTCTCAACTTGTGGGGACCG
USP9Y	F: GACCCTTGTGTATCAGCAGCATTTC
	R: GCTGCCCAGTAATGACTAAGTCCA
USP9X	F: CCCAGTGCAAAGCAATGAAACG
	R: TGCTCTCTTCCATTTCTATCTGCTGC

Supplementary movie 1. Spontaneous beating colonies of cardiomyocytes
